# A Modified Nerve Preservation Technique in Radical Hysterectomy: Three-Dimensional Precise Dissection of Paracolpium

**DOI:** 10.1055/s-0044-1800979

**Published:** 2024-12-20

**Authors:** Jing Na, Ya Li, Jun Wang, Shichao Han

**Affiliations:** 1 Department of Obstetrics and Gynecology, The Second Affiliated Hospital of Dalian Medical University, Dalian, Liaoning Province, People's Republic of China

Radical hysterectomy is the gold standard surgical treatment for early-stage cervical cancer. 1 However, despite its efficacy in cancer control, this procedure often results in significant postoperative complications. Many patients experience prolonged urinary dysfunction, such as urinary retention, incontinence, and frequent urinary tract infections, as well as gastrointestinal issues, including constipation and defecation disorders. These complications can severely diminish the quality of life, posing long-term challenges to patients' well-being.

Given these concerns, there is a growing need for surgical techniques that not only achieve the oncological objectives of radical hysterectomy but also minimize damage to the autonomic nerves responsible for bladder and rectal function. The preservation of these functions is crucial for improving postoperative recovery and long-term quality of life.

In response to this need, Querleu and Morrow 2 proposed the type C1 nerve-sparing radical hysterectomy in 2008, which focuses on preserving autonomic nerve function within the pelvis. This innovative approach aims to reduce the incidence of urinary and gastrointestinal complications by carefully sparing the pelvic nerves during surgery.

The technique was further refined in 2017, 3 incorporating advancements in surgical anatomy and dissection techniques. As a result, the type C1 nerve-sparing surgery has gained recognition and has been endorsed by several clinical guidelines, including those of the National Comprehensive Cancer Network, 4 as a recommended approach for the surgical management of early-stage cervical cancer.

The adoption of nerve-sparing techniques, such as the C1 approach, represents a significant advancement in gynecologic oncology, offering a balance between effective cancer control and the preservation of essential pelvic functions. As more surgeons adopt these methods, we can anticipate improved outcomes for patients, not only in terms of survival but also in their overall quality of life after surgery.

Performing the type C1 nerve-sparing radical hysterectomy, a procedure known for its exceptionally high level of difficulty, presents significant challenges. This complexity is a major concern for many surgeons. Successfully implementing and executing this surgery requires achieving both an adequate extent of resection and the preservation of the nerves that innervate the bladder. This task is critically important yet incredibly challenging. The primary difficulty lies in accurately identifying and preserving these autonomic nerves without causing any damage, while also ensuring that the surgical resection is sufficiently extensive for effective cancer control.

The type C1 surgical procedure focuses on preserving pelvic autonomic nerve function, particularly the inferior hypogastric plexus (IHP), which is formed by the convergence of the pelvic splanchnic nerves and the hypogastric plexus. 5 Although the specific locations and pathways of these nerves are extensively documented, there is a notable lack of detailed, step-by-step surgical methods for effectively implementing this nerve-sparing approach.

**Fig. 1:**
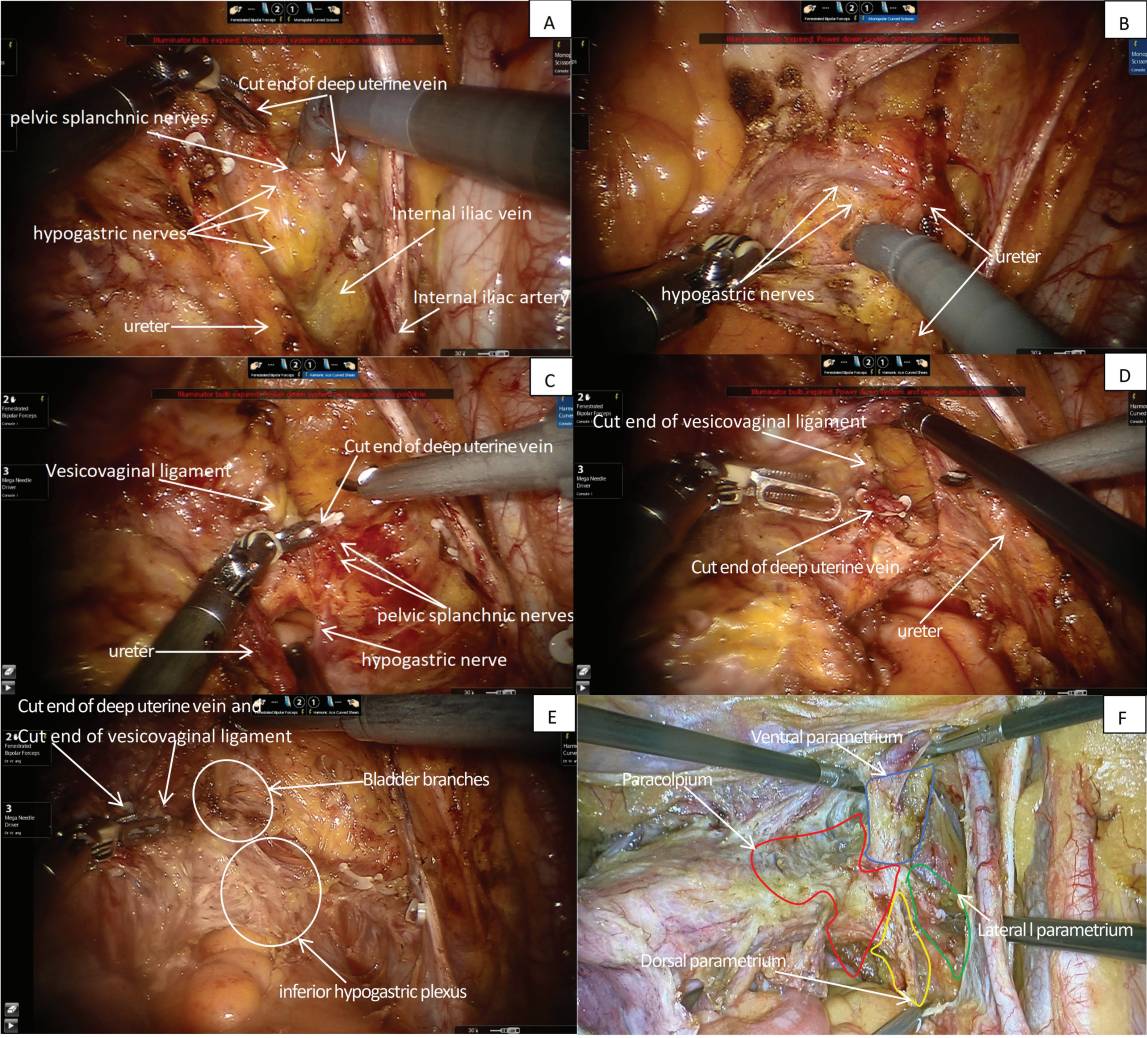
( **A, B** ) Images showing the location of the pelvic autonomic nerves and the hypogastric plexus. ( **C** ) Image showing the convergence of the pelvic autonomic nerves and the hypogastric plexus at the dorsolateral aspect of the deep uterine veins within the paracolpium. ( **D** ) Image showing the vascular plane formed by the deep uterine veins and the bladder venous plexus, which is located within the vesicovaginal ligament and drains into the deep uterine veins. ( **E** ) Image showing the neural plane located posterior to the vascular plane within the paracolpium and the bladder branches of IHP. ( **F** ) Image showing the paracolpium as a three-dimensional structure composed of contributions from the ventral, lateral, and dorsal parametrium. IHP, inferior hypogastric plexus.

Through detailed anatomical studies of cervical cancer surgery, we have discovered that the pelvic splanchnic nerves are located posterior to the deep uterine veins ( Fig. 1A ), while the hypogastric plexus is found within the retroperitoneal tissue, posterior to the ureter ( Fig. 1A, B ). Consequently, their convergence does not occur at the parametrium but rather within the paracolpium ( Fig. 1C ). The paracolpium contains a complex network of venous plexuses, making the precise, bloodless dissection of the IHP while preserving the bladder branches of the pelvic autonomic nerves a significant challenge. Therefore, a comprehensive understanding of the paracolpium's anatomy is crucial for successful implementation.

Three-dimensional anatomical dissection of the paracolpium ( Fig. 1F ) reveals its composition as follows: the deep uterine veins and the surrounding lymphatic adipose tissue of lateral parametrium, the uterosacral ligaments of dorsal parametrium, and the vesicovaginal ligament of ventral parametrium. The vesicovaginal ligament houses the bladder venous plexus, which drains into the deep uterine veins. 6 7 Consequently, the ventral aspect of the paracolpium comprises a vascular plane formed by the deep uterine veins and the bladder venous plexus. The posterior aspect of this vascular plane consists of a neural plane formed by the hypogastric plexus and the pelvic splanchnic nerves, along with the nerve branches originating from this plexus. The posterior aspect of the neural plane is further defined by the sacral ligaments.

To achieve optimal surgical outcomes, the vascular plane should be mobilized medially as a cohesive unit ( Fig. 1D, E ), thereby exposing the underlying neural plane ( Fig. 1E ). The goal of this maneuver is to preserve the bladder branches by carefully transecting the medial branches of the IHP . This approach not only aims to minimize nerve damage but also ensures that the bladder's autonomic innervation is retained, thereby improving patient outcomes and preserving functional capabilities.
